# A Rare Case of Neglected Fifth Metatarsophalangeal Joint Dislocation

**DOI:** 10.7759/cureus.28636

**Published:** 2022-08-31

**Authors:** Aditya Sharma, Venkata Sai Harshabhargav Chenna, Hemanth Kumar Reddy Pethari, Pradeep Pentapurthy, Sri Ram Pureti, Anuj Gupta

**Affiliations:** 1 Orthopedics and Trauma, Max Super Specialty Hospital, Vaishali, IND; 2 Medicine, St. Martinus University, Willemstad, CUW; 3 Orthopedics and Spine, Triveni Ortho and Spine Center, Delhi, IND; 4 Spine Surgery, Max Super Specialty Hospital, Vaishali, IND

**Keywords:** neglected, foot, metatarsophalangeal joint, dislocation, lesser toe

## Abstract

The dislocation of the metatarsophalangeal joint of lesser toes is a rare entity. There is a dearth of literature on the same. Also, there is no case described for neglected fifth metatarsophalangeal dislocation in the literature. We present a case of neglected lesser toe dislocation, its natural course, and its outcome after surgical management. Our patient is an eight-year-old child with a neglected dislocation of the fifth metatarsophalangeal joint two years back. The patient did not seek treatment because he has no problem walking. Gradually, there is an abnormal growth of the metatarsal which causes pressure soreness and difficulty walking. The patient was managed surgically with open reduction and K-wire fixation with good long-term results.

The dislocation of fifth metatarsophalangeal dislocation is rare and may not cause difficulty in walking due to less weight-bearing. But prompt treatment is necessary, especially in children as the bones have remaining growth potential and may lead to abnormal bone growth.

## Introduction

The forefoot is comprised of the great toe and lesser toes along with their respective metatarsals. Traumatic dislocation of isolated metatarsophalangeal joints of lesser toes is very uncommon [1,2]. The isolated traumatic dislocation of the fifth metatarsophalangeal joint is even rarer. However, if left untreated, it may lead to significant limitations on the functioning of the patient [3]. There are a few cases described in the literature for irreducible dislocation of the fifth metatarsophalangeal joint [1,4]. However, to the best of our knowledge, there is no case described for neglected fifth metacarpophalangeal dislocation in the literature. We present a case of the same, its natural course in an eight-year-old child, and its management.

## Case presentation

**Figure 1:**
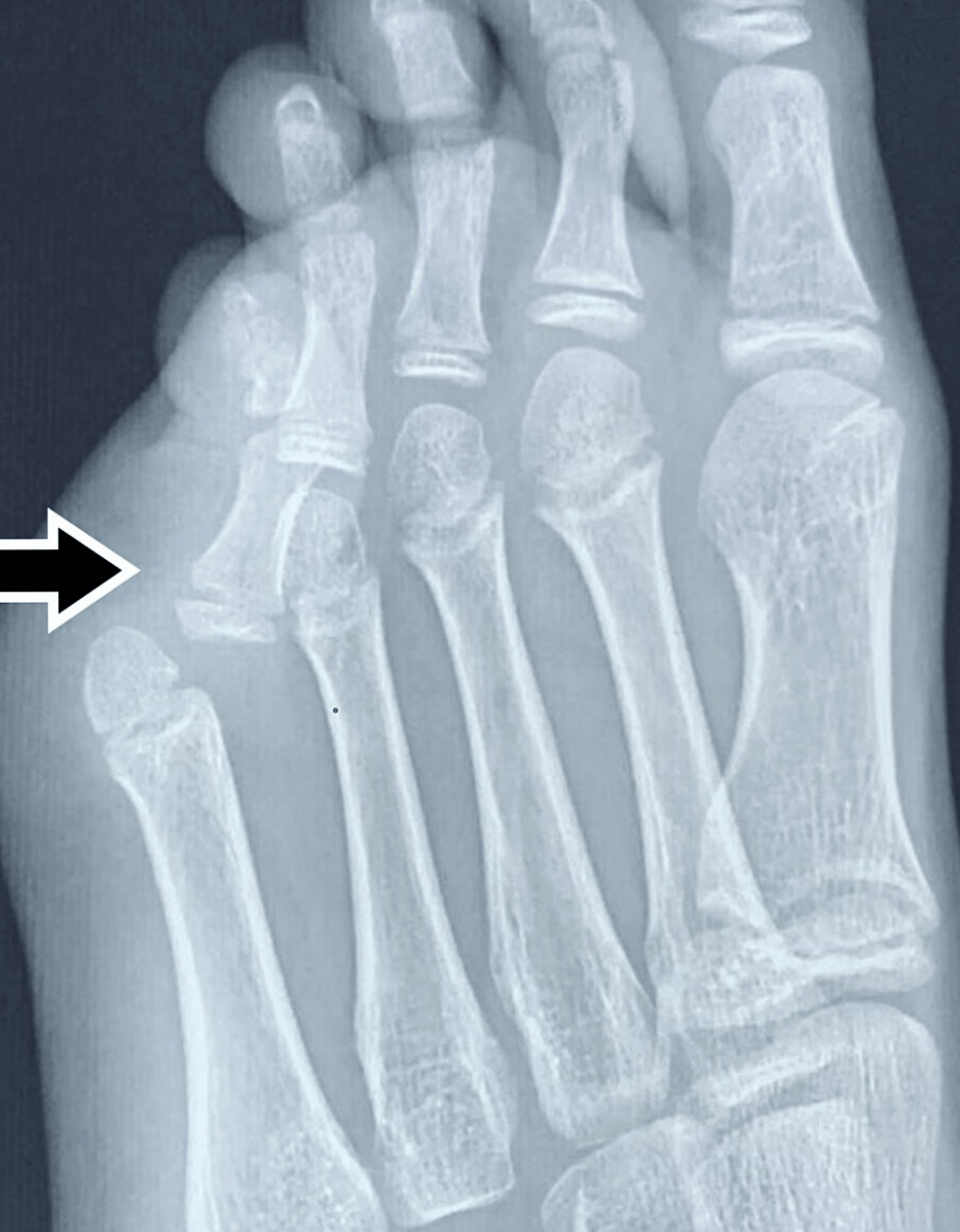
Pre-operative (at the time of presentation, two years after injury) x-ray showing dislocation of the fifth metatarsophalangeal joint

**Figure 2:**
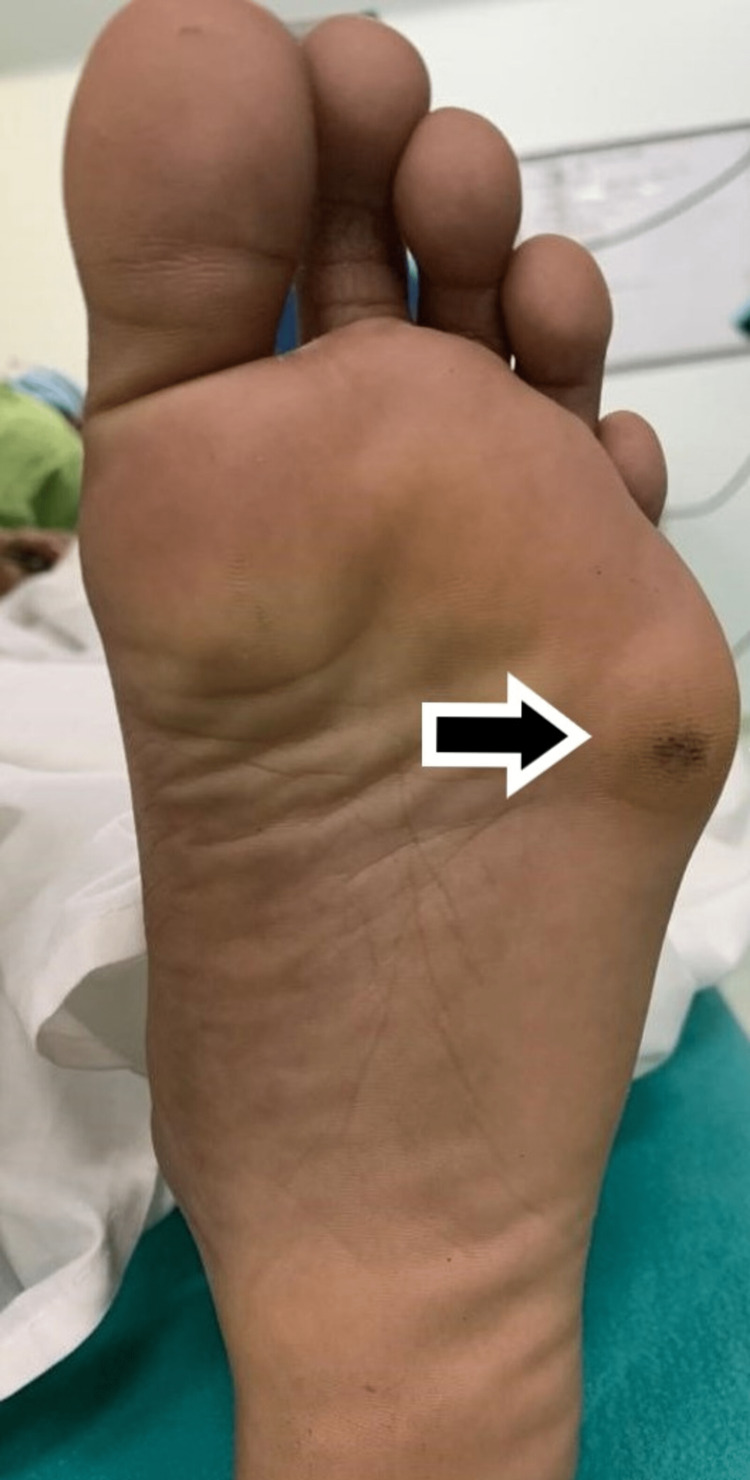
Clinical photograph showing callosity at plantar aspect of foot

An eight-year-old child presented to us with complaints of pain in his left foot. There was a history of injury to his left foot two years back. He was diagnosed with dislocation of the fifth metatarsophalangeal joint but refused surgery. After that, the child started walking on subsidence of pain and took no further treatment. According to history, there was no swelling at the time of injury. But, when the patient presented to us, there was callosity seen at the plantar aspect of the foot and the distal end of the fifth metatarsal can be appreciated in that area (Figures 1, 2). There was no pain on palpation and the ends of metatarsophalangeal joints can be palpated. The proximal end of the proximal phalanx can be palpated dorsally. There were no records or x-ray available of the patient at the time of injury.

**Figure 3:**
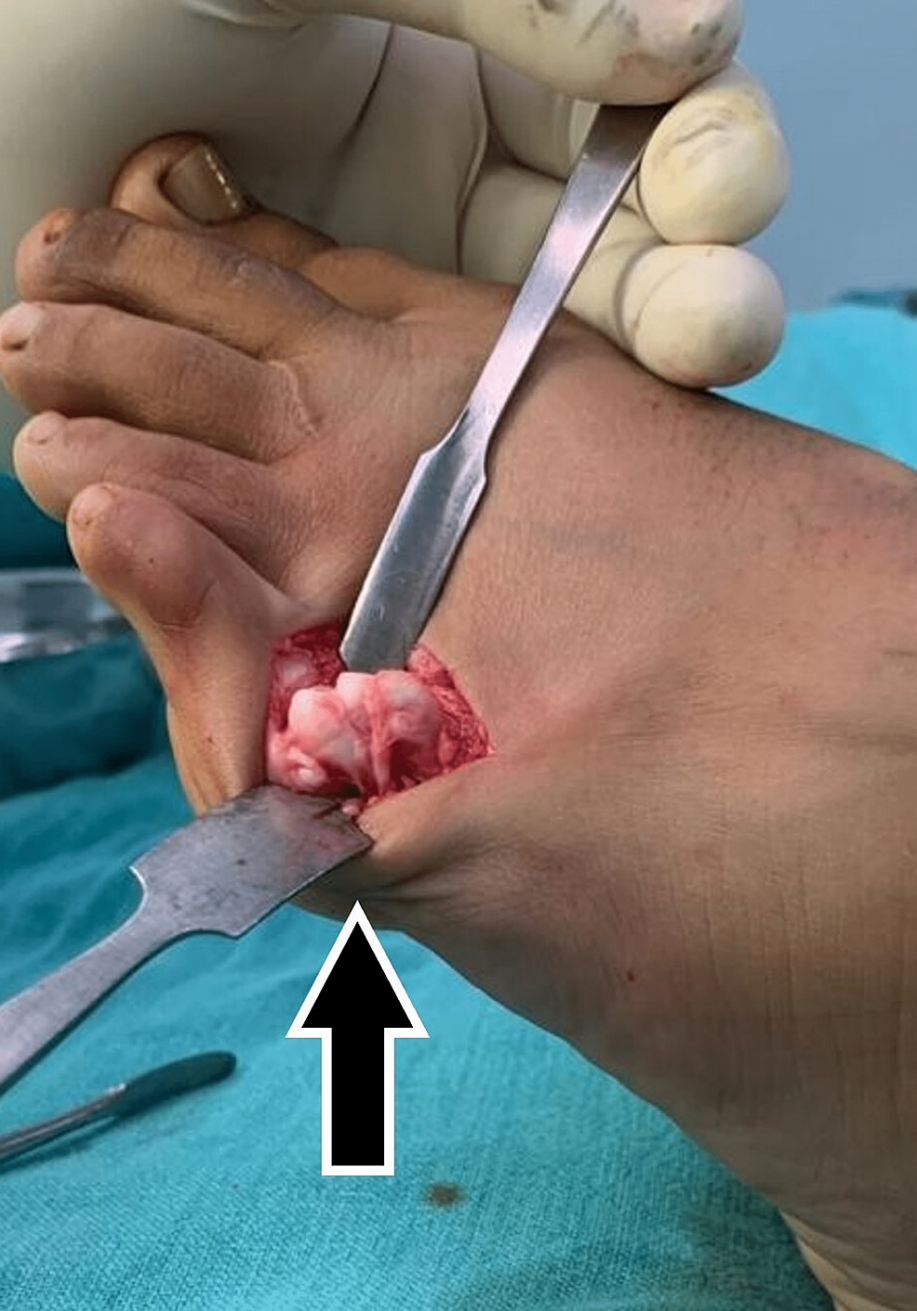
Dorsal incision showing distal end of fifth metatarsal with bone growth at epiphysis

It was decided to manage it surgically with open reduction and fixation with k wire. The patient was prepared, the incision is given on the dorsal aspect at the involved joint, and the end of the fifth metatarsal was identified (Figure 3).

**Figure 4:**
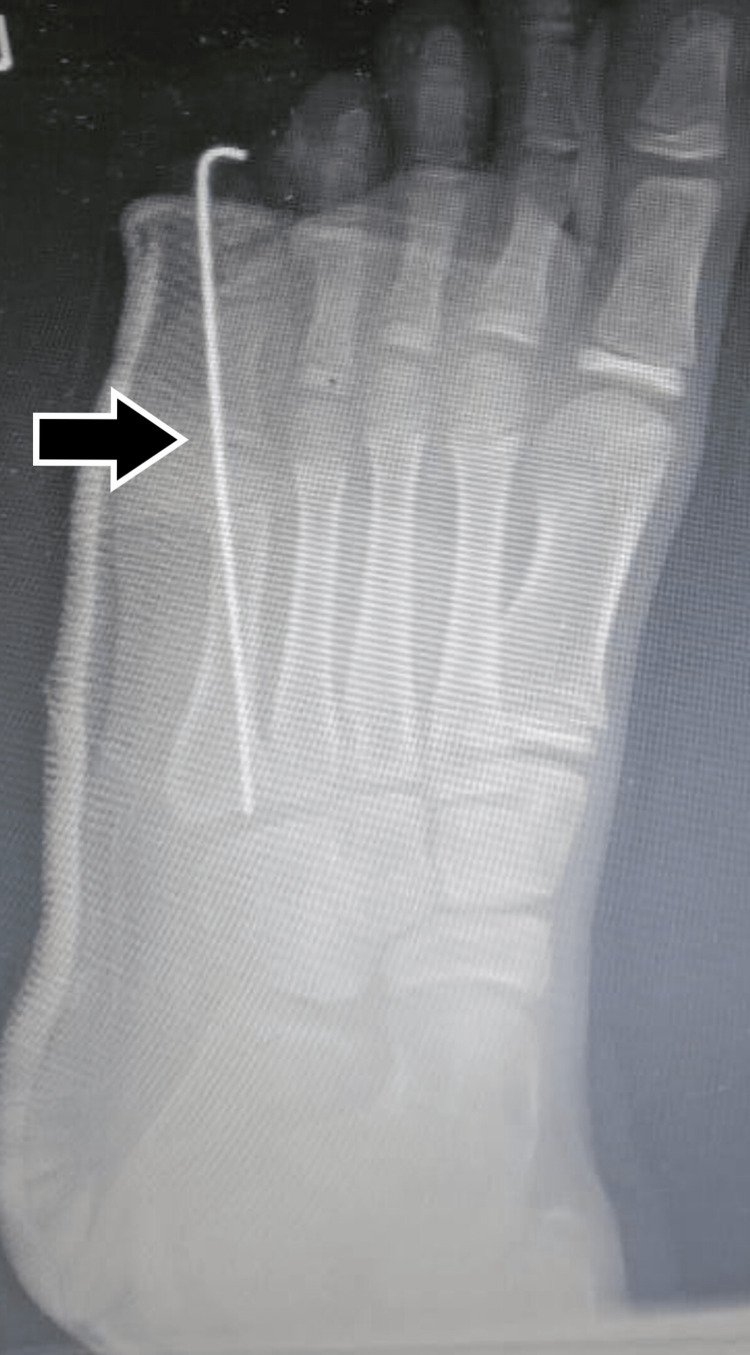
Immediate post-operative x-ray with k-wire

The growth of epiphysis could be appreciated which was responsible for swelling and callosity formation after two years of injury. The distal end of the proximal phalanx was also cleared and the joint was reduced. The k-wire was used to hold the reduction in place (Figure 4).

**Figure 5:**
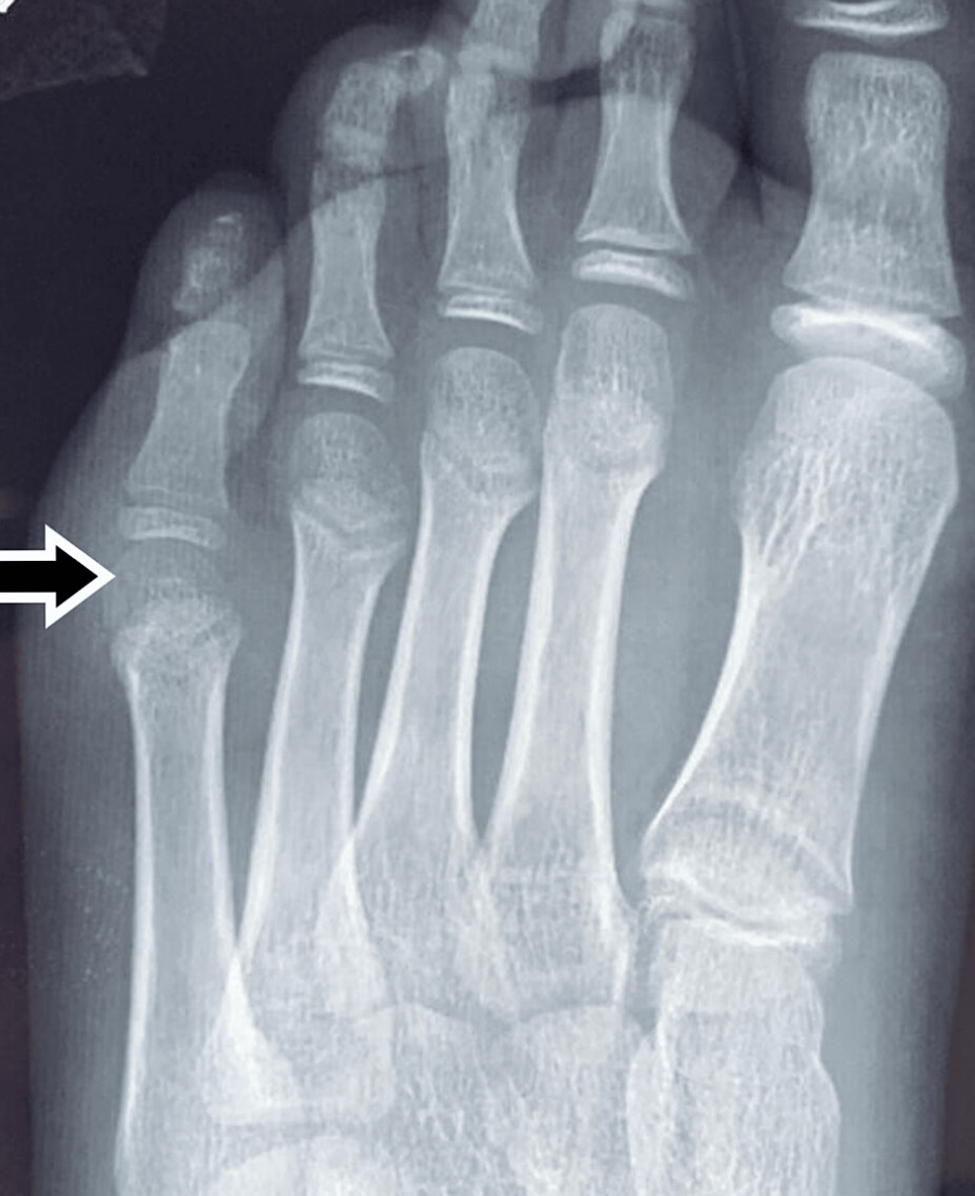
X-ray after two years of follow-up with maintained reduction

A plaster cast was applied to support the fixation and the limb was immobilized for six weeks. Thereafter, the cast was removed, the k-wire too was removed, and the patient was asked to bear full weight. On further follow-up up to two years, the callosity subsided gradually and the patient has good relief in pain. On x-rays, the reduction was maintained satisfactorily (Figure 5).

However, the range of motion (ROM) of the fifth metatarsophalangeal joint was adequate but not full as of the opposite limb. Still, the patient has no limitation in any activity.

## Discussion

The dislocation of the lesser metatarsophalangeal joint is a rare entity and the involvement of the fifth metatarsophalangeal joint is even rarer. Considering the biomechanics of weight-bearing on the foot, the great toe and its metatarsophalangeal joint have to bear maximum weight compared to lesser joints, but there is definitely a role for lesser metatarsophalangeal joints in weight-bearing [5]. Even the fifth metatarsophalangeal joint contributes some weight-bearing. Hence, disorders of this joint can limit the functionality of the foot. Most often, the direction of dislocation is dorsal due to forced hyperextension of the joint due to injury [4]. In our case too, the direction of displacement is dorsal. Due to hyperextension, the plantar capsule and planar plate are torn off the neck and get interposed between the parts of the joint preventing reduction [6]. There are rare cases reported too in the literature where the direction of displacement is plantar with a possible mechanism of injury as hyperflexion [2,7].

The dislocation might cause some or no problems in adults as the percentage of weight-bearing is low compared to other joints. But in children the scenario is different. In our patient who is eight years old, the initial injury poses not much problem except pain which subsided gradually and the patient experienced no limitation in any function. But gradually, due to the remaining growth potential of the physis, there was the growth of physis which in turn led to an increase in length of the bone and impingement of ends of the bone on the plantar aspect. This leads to the formation of callosity and ulcers in that area which became painful for weight-bearing to the patient. In our view and to the best of our knowledge, this is the first case report which describes this as one of the indications of early management of dislocation of the fifth metatarsophalangeal joint.

Most fifth metatarsophalangeal joint dislocations can be reduced by means of closed reduction and this is possible only in acute cases and where there is no incarceration of soft tissues [8]. But cases that are neglected or there are interposed soft tissues warrant a surgical reduction. In surgical management, the dorsal approach is the best approach for dorsal dislocation [9]. However, few surgeons prefer to approach via plantar incision as they feel it gives a better approximation to deep transverse intermetatarsal ligament and plantar plate [9]. In our patient, we used the dorsal approach and were comfortably able to make out the ends of the joint which in turn made us to reduced and fix the joint appropriately.

The range of motion is compromised in cases of neglected dislocations. In our case, we achieved a good range of motion despite the fact that the time gap between surgical intervention and the time of injury was two years. Although there was some terminal restriction of the joint motion, there was no limitation in the activities of the patient. The reason for a good range of motion despite the late reduction of the joint could possibly be the young age of the patient and early removal of the k-wire and mobilization of the joint.

## Conclusions

The dislocation of the fifth metatarsophalangeal joint is rarer compared to the dislocation of other metatarsophalangeal joints of the foot. Even if the weight-bearing is less, the joint requires early reduction, especially in children. In adults, the neglected dislocation may not cause any limitation but in children, due to remaining growth potential, the neglected dislocation may present with callosity and pressure ulcers a few years after the injury.
